# Aptamer-Based Switching System for Communication of Non-Interacting Proteins

**DOI:** 10.3390/bios14010047

**Published:** 2024-01-16

**Authors:** Younghyeon Kim, Daehan Nam, Eun Sung Lee, Seokjoon Kim, Byung Seok Cha, Ki Soo Park

**Affiliations:** Department of Biological Engineering, College of Engineering, Konkuk University, Seoul 05029, Republic of Korea; qaz2432@konkuk.ac.kr (Y.K.); nam5192001@konkuk.ac.kr (D.N.); afish94@konkuk.ac.kr (E.S.L.); ghjghy@konkuk.ac.kr (S.K.); cbs934@konkuk.ac.kr (B.S.C.)

**Keywords:** biological network, protein, aptamer, CRISPR/Cas, T7 RNA polymerase

## Abstract

Biological macromolecules, such as DNA, RNA, and proteins in living organisms, form an intricate network that plays a key role in many biological processes. Many attempts have been made to build new networks by connecting non-communicable proteins with network mediators, especially using antibodies. In this study, we devised an aptamer-based switching system that enables communication between non-interacting proteins. As a proof of concept, two proteins, Cas13a and T7 RNA polymerase (T7 RNAP), were rationally connected using an aptamer that specifically binds to T7 RNAP. The proposed switching system can be modulated in both signal-on and signal-off manners and its responsiveness to the target activator can be controlled by adjusting the reaction time. This study paves the way for the expansion of biological networks by mediating interactions between proteins using aptamers.

## 1. Introduction

In nature, interactions between DNA, RNA, and proteins form the basis for a complex network that plays an important role in various biological processes [1]. Many studies have been conducted to understand the mechanisms underlying these interactions [2]. Furthermore, attempts have been made to design new synthetic circuits for applications such as therapeutics, drug delivery, and cell imaging [3,4,5,6]. In principle, reaction pathways can be created using various mediators to mimic many in vivo reactions, enabling communication between non-interacting proteins that can trigger chain reactions [7]. However, network studies have mostly been limited to proteins and their antibodies [8]. Therefore, we introduce a different methodology to build this network which is based on the use of aptamers as mediators.

Compared with antibodies, which are typically used to build networks with proteins, aptamers have several advantages [9]. Aptamers are short, single-stranded nucleic acids that can bind to specific target substances, such as small molecules, proteins, exosomes, and cells, with high affinity [10]. In contrast to antibodies, aptamers are discovered by an in vitro screening methodology called systematic evolution of ligands by exponential enrichment (SELEX) and are synthesized by an in vitro chemical process [11,12]. Thus, their production is relatively rapid and cost-effective, guaranteeing consistent analytical performance [13]. Because of the advantageous features of aptamers, many diagnostic and therapeutic strategies have been developed [14]. The representative examples are found in the systems using aptamers to recognize the specific target molecules, such as small molecules and proteins, which can be operated in signal-on and -off manners [15,16]. The signal-on system is defined as the one that increases the signal by the presence of target molecules, while the signal-off system is defined as the one that decreases the signal by the presence of target molecules. However, the use of aptamers as mediators to create new networks for biological processes in which two non-interacting proteins are connected has not been reported.

CRISPR-Cas13a has been utilized in a variety of applications, including molecular diagnostics, gene therapy, gene editing, and RNA imaging [17,18,19,20,21], because it catalyzes cis-cleavage reactions on a specific single-stranded RNA (ssRNA) sequence containing a protospacer flanking site (PFS), followed by indiscriminate trans-cleavage reactions on ssRNA sequences [22,23]. T7 RNA polymerase (T7 RNAP) is a DNA-dependent RNA polymerase that produces RNA transcripts from template DNA with a T7 promoter and has been used in many studies including molecular diagnostics and RNA imaging [24,25,26,27]. Based on their own functions, these two proteins can be linked to each other by a standard process in which T7 RNAP-catalyzed production via RNA transcription activates Cas13a, leading to efficient cis- and trans-cleavage reactions [28,29]. This format has been utilized for the detection of various biomarkers [30]. However, other Cas13a and T7 RNAP connection mechanisms during which the Cas13a-catalyzed reaction regulates the activities of T7 RNAP have not been reported. Therefore, the interaction of Cas13a with the subsequent T7 RNAP-catalyzed reaction must be mediated to construct a more complex network.

In this study, we utilized an aptamer as a mediator for the communication between two non-interacting proteins based on their intrinsic functions and designed a fluorescence switching system that operates in both signal-on and signal-off manners. CRISPR-Cas13a and T7 RNAP, which have unique catalytic properties and versatile uses in different fields, were selected as model proteins. Specifically, a T7 RNAP-specific RNA aptamer (T7 RNAP aptamer) with an inhibitory activity against T7 RNAP was used as a mediator to link Cas13a with T7 RNAP. Furthermore, we designed a signal-on system in which activated Cas13a degrades the T7 RNAP aptamer, generating a high fluorescence signal based on effective T7 RNAP-catalyzed transcription. We also designed a signal-off system in which a blocker RNA was introduced into a T7 RNAP aptamer such that the activated Cas13a degrades the blocker RNA and the T7 RNAP aptamer inhibits the T7 RNAP-catalyzed transcription, generating a negligible fluorescence signal. The proposed switching system was thoroughly validated by confirming the trans-cleavage reaction of Cas13a on both the T7 RNAP aptamer and the blocker RNA, the inhibitory ability of the T7 RNAP aptamer against T7 RNAP, and the operation of the integrated system. Notably, an aptamer-based switching system that was constructed for the communication between Cas13a and T7 RNAP in a different manner could allow for the construction of complex logic circuits.

## 2. Materials and Methods

### 2.1. Materials and Reagents

All oligonucleotides (Table S1) used in this study were purchased from Bionics (Seoul, Republic of Korea) and Integrated DNA Technologies (Coralville, IA, USA). T7 RNA polymerase, T7 RNA polymerase buffer, and RNase inhibitors were obtained from Enzynomics (Daejeon, Republic of Korea). The HiScribe T7 High Yield RNA synthesis kit, Monarch RNA cleanup kit, DNase I, DNase I reaction buffer, NEBuffer™ r2.1, and rNTP were purchased from New England Biolabs (Ipswich, MA, USA). Plates with 384 black wells were purchased from SPL Life Sciences (Pocheon, Republic of Korea). To1-3PEG-Biotin Fluorophore (To1-biotin) was obtained from Applied Biological Materials (Richmond, BC, Canada). LbuCas13a was purchased from SignalChem Diagnostics (St. Louis, MO, USA).

### 2.2. In Vitro Transcription of crRNA

The crRNA used in this experiment was synthesized via in vitro transcription. The reaction mixture consisted of 0.75× reaction buffer, 1 μM crRNA template, 7.5 mM rATP, 7.5 mM rUTP, 7.5 mM rGTP, 7.5 mM rCTP, 1.5 μL T7 RNA polymerase, and nuclease-free water (up to 20 μL). After the reaction mixture was incubated for 16 h at 37 °C, it was treated with 0.04 U/μL DNase I, 1× DNase I reaction buffer, and nuclease-free water (up to 100 μL). Subsequently, it was incubated for 15 min at 37 °C. Finally, the RNA transcription products were purified using a Monarch RNA cleanup kit and their concentrations were quantified by measuring the absorbance at a wavelength of 260 nm using SpectraMax iD5 Multi-Mode Microplate Reader (Molecular Devices, San Jose, CA, USA), which was stored at −20 °C prior to use.

### 2.3. Cas13a-Catalyzed Trans-Cleavage Reaction

The experiments to confirm the T7 RNAP aptamer cleavage based on Cas13a were performed in a reaction mixture containing 1× NEBuffer™ r2.1, 20 nM LbuCas13a, 10 nM crRNA, 300 nM T7 RNAP aptamer or 300 nM blocker RNA, 1 nM target RNA activator (miR-141), and nuclease-free water (up to 20 μL). After the reaction mixture was incubated for 30 min at 37 °C and for 10 min at 80 °C for the inactivation of Cas13a, the results were analyzed by electrophoresis at 135 V for 30 min on a 3% agarose gel.

### 2.4. T7 RNAP-Catalyzed Transcription Reaction

The experiments were performed in a reaction mixture containing 1× T7 RNA polymerase buffer (40 mM Tris-HCl, 10 mM MgCl_2_, 10 mM DTT, 2 mM spermidine, pH 7.9), 0.25 mM each rNTP, 100 nM To1-biotin, 10 nM Mango template, 10 nM T7 promotor, 100 nM T7 RNAP aptamer, 300 nM blocker RNA (only for signal-off system), 0.8 U/μL RNase inhibitor, 0.5 U/μL T7 RNA polymerase, and nuclease-free water (up to 40 μL). After the reaction mixture was incubated for 30 min at 37 °C, it was transferred to a 384-black well plate and the fluorescence signals of To1-biotin were measured at excitation and emission wavelengths of 507 and 547 nm, respectively, at 25 °C using a microplate reader [31]. For the measurement of the fluorescence spectra, the emission wavelength range was set to 520–620 nm, with an excitation wavelength of 480 nm.

### 2.5. Operation of the Aptamer-Based Signal-On System

The reaction mixture was composed of 1× NEBuffer™ r2.1 (50 mM NaCl, 10 mM Tris-HCl, 10 mM MgCl_2_, 100 µg/mL recombinant albumin, pH 7.9 at 25 °C), 5 nM LbuCas13a, 2.5 nM crRNA, 900 nM T7 RNAP aptamer, various concentrations of target RNA activator (miR-141), and nuclease-free water (up to 20 μL), which was incubated for 30 min at 37 °C and for 10 min at 80 °C for the inactivation of Cas13a. Subsequently, 4 μL of the reaction mixture was treated with 1× T7 RNA polymerase buffer (40 mM Tris-HCl, 10 mM MgCl_2_, 10 mM DTT, 2 mM spermidine, pH 7.9), 0.25 mM each rNTP, 100 nM To1-biotin, 10 nM Mango template, 10 nM T7 promoter, 0.8 U/μL RNase inhibitor, 0.5 U/μL T7 RNA polymerase, and nuclease-free water (up to 40 μL). After the reaction mixture was incubated for 30 min at 37 °C, it was transferred to a 384-black well plate and the fluorescence signals of To1-biotin were measured at excitation and emission wavelengths of 507 and 547 nm, respectively, at 25 °C using a microplate reader.

### 2.6. Operation of the Aptamer-Based Signal-Off System

The reaction mixture was composed of 1× NEBuffer™ r2.1 (50 mM NaCl, 10 mM Tris-HCl, 10 mM MgCl_2_, 100 µg/mL recombinant albumin, pH 7.9 at 25 °C), 5 nM LbuCas13a, 2.5 nM crRNA, 1.5 μM blocker RNA, various concentrations of target RNA activator (miR-141), and nuclease-free water (up to 20 μL), which was incubated for 30 min at 37 °C and for 10 min at 80 °C for inactivation of Cas13a. Subsequently, 4 μL of the reaction mixture was treated with 1× T7 RNA polymerase buffer (40 mM Tris-HCl, 10 mM MgCl_2_, 10 mM DTT, 2 mM spermidine, pH 7.9), 0.25 mM each rNTP, 100 nM To1-biotin, 10 nM Mango template, 10 nM T7 promoter, 90 nM T7 RNAP aptamer, 0.8 U/μL RNase inhibitor, 0.5 U/μL T7 RNA polymerase, and nuclease-free water (up to 40 μL). After the reaction mixture was incubated for 10 min at 37 °C, it was transferred to a 384-black well plate and the fluorescence signals of To1-biotin were measured at excitation and emission wavelengths of 507 and 547 nm, respectively, at 25 °C using a microplate reader.

## 3. Results and Discussion

### 3.1. Working Principle of the Aptamer-Based Switching System for the Communication of Cas13a with T7 RNAP

**Figure 1:**
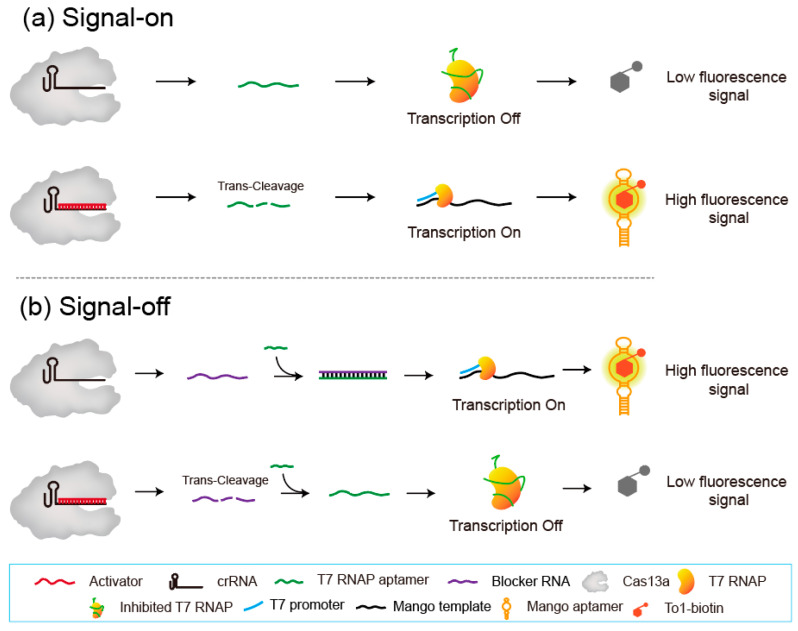
Schematic illustration of the aptamer-based switching system for the communication of Cas13a and T7 RNAP, which operated in both (**a**) signal-on and (**b**) signal-off manners.

We designed a signal-on system for the communication between Cas13a and T7 RNAP using a T7 RNAP aptamer. Figure 1a shows that the absence of an RNA activator deactivates the Cas13a-crRNA complex and the intact T7 RNAP aptamer binds to T7 RNAP and inhibits its transcriptional activity, resulting in a very low unbound fluorescence signal for To1-biotin. In contrast, the presence of an RNA activator enables the Cas13a-crRNA complex to exert intrinsic trans-cleavage activities on the T7 RNAP aptamer and thus the active T7 RNAP generates a light-up Mango aptamer via effective transcription, emitting a high fluorescence signal upon complexation with To1-biotin. Furthermore, a signal-off system was devised by rationally designing a blocker RNA partially complementary to the T7 RNAP aptamer (Figure 1b). In the absence of an RNA activator, the Cas13a-crRNA complex becomes inactive and the blocker RNA hybridizes with the T7 RNAP aptamer, preventing the T7 RNA aptamer from binding to T7 RNAP. Thus, active T7 RNAP catalyzes the transcription and generates a light-up Mango aptamer, leading to a strong fluorescent signal upon complexation with To1-biotin. In contrast, the Cas13a-crRNA complex activated by the presence of an RNA activator degrades the blocker RNA due to its indiscriminate trans-cleavage activities and thus the free T7 RNAP aptamer inhibits the T7 RNAP activity, resulting in the negligible, unbound fluorescence signal of To1-biotin. Cas13a and T7 RNAP were connected using T7 RNAP aptamers as mediators, which were operated in both signal-on and -off manners by generating a high and low fluorescence signal in the presence of an RNA activator, respectively. Considering the clinical significance of miRNAs in cancer development, proliferation, and metastasis, miRNA-141 was chosen as the RNA activator.

### 3.2. Feasibility of the Signal-On System

**Figure 2:**
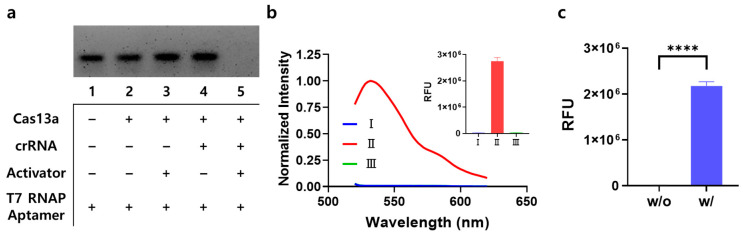
Feasibility of the signal-on system. (**a**) Agarose gel electrophoresis results confirming the trans-cleavage reaction of Cas13a on the T7 RNAP aptamer. Lane 1: T7 RNAP aptamer (300 nM), lane 2: lane 1 + Cas13a (20 nM), lane 3: lane 2 + RNA activator (miR-141; 1 nM), lane 4: lane 2 + crRNA (10 nM), and lane 5: lane 4 + RNA activator (1 nM). (**b**) Normalized fluorescence emission spectra and fluorescence intensity data proving the T7 RNAP aptamer-mediated inhibition of T7 RNAP-catalyzed transcription. T7 RNAP-catalyzed transcription in each case was performed as follows: (I) without the T7 promoter, (Ⅱ) without the T7 RNAP aptamer, and (Ⅲ) with all elements added. Fluorescence intensities were normalized using the maximum value. (**c**) Fluorescence intensities of the signal-on system, which relies on the combination of Cas13a with T7 RNAP using the T7 RNAP aptamer as mediator. “w/o” and “w/” indicate without and with target RNA activator, respectively. Unpaired *t*-test: **** *p* < 0.0001. All tests were performed with three technical replicates.

To verify the feasibility of the proposed signal-on system, we conducted a step-by-step experiment using miR-141 as an RNA activator. First, the trans-cleavage activity of Cas13a against the T7 RNAP aptamer was investigated using gel electrophoresis. Figure 2a shows that the gel band corresponding to the T7 RNAP aptamer disappeared when the Cas13a-crRNA complex was present with the RNA activator, confirming the effective degradation of the T7 RNAP aptamer by the trans-cleavage activity of Cas13a. Subsequently, T7 RNAP-catalyzed transcription and its inhibition by the T7 RNAP aptamer were evaluated by measuring the fluorescence emission spectrum of To1-biotin (Figure 2b). As expected, T7 RNAP did not initiate transcription in the absence of the T7 promoter, generating a very low fluorescence signal (I), whereas T7 RNAP-catalyzed transcription to produce a Mango aptamer occurred in the presence of the T7 promoter and the absence of the T7 RNAP aptamer, emitting a high fluorescence signal upon complexation with To1-biotin (II). Notably, the application of the T7 RNAP aptamer resulted in a negligible fluorescence signal, proving that it effectively inhibited T7 RNAP (III). Finally, we integrated two separate reactions catalyzed by Cas13a and T7 RNAP and checked whether the linked system could be operated by an RNA activator in a signal-on manner. The results in Figure 2c show that the presence of the RNA activator enables Cas13a to catalyze indiscriminate trans-cleavage reactions on the T7 RNAP aptamer. Thus, effective transcription catalyzed by T7 RNAP occurred, generating a high fluorescence signal of To1-biotin upon complexation with the Mango aptamer, which differs from the negligible fluorescence signal in the absence of an RNA activator. Overall, these results demonstrate that the two reactions catalyzed by Cas13a and T7 RNAP were combined to create a new network.

To improve the performance of the aptamer-based switching system, further optimizations were performed by investigating the effects of Cas13a concentration, crRNA concentration, and transcription time. As shown in Figure S1a, Supplementary Material, the Cas13a concentration was determined to be 5 nM because a distinguishable fluorescence signal was generated at 5 nM. The optimal crRNA concentration was also screened using the optimized Cas13a concentration. The results in Figure S1b show that crRNA at concentrations below 5 nM yielded a sufficiently higher signal than that at concentrations higher than 10 nM. And a crRNA concentration of 2.5 nM was selected for further experiments because it matched the recent result that the optimal ratio of Cas13a:crRNA is 2:1 [32]. Furthermore, 10 min was chosen as the optimal time for T7 RNAP-catalyzed transcription because the signal plateaued at a transcription time above 10 min (Figure S1c).

### 3.3. Performance of the Signal-On System

**Figure 3:**
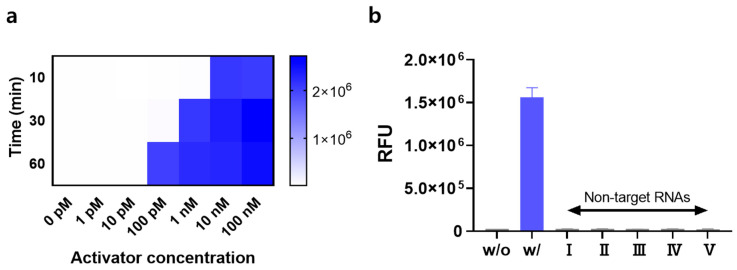
Performance of the signal-on system. (**a**) Fluorescence intensities measured at various RNA activator concentrations (0–100 nM) and Cas13 reaction times ranging from 10 to 60 min. (**b**) Fluorescence intensities in the presence of target RNA activator and non-target RNAs (10 nM). “w/o” and “w/” indicate without and with target RNA activator, respectively. Non-target RNAs I–V are miR-21, miR-155, miR-182, miR-373, and miR-375, respectively. All tests were performed with three technical replicates.

We conducted experiments to verify the detection performance of the signal-on system under optimal conditions. First, the sensitivity was evaluated by measuring the signal in the presence of the target RNA activator at various concentrations. When the concentration of the RNA activator increased, a strong signal was observed. Interestingly, the detection range of the RNA activator was adjusted by varying the reaction time with Cas13a. Figure 3a shows that a high fluorescence signal started to form at an RNA activator concentration of 1 nM and Cas13a reaction time of 30 min, whereas the lowest concentration of the RNA activator that initiated the signal-on system changed to 100 pM or 10 nM based on the increase or decrease in the reaction time to 60 min or 10 min, respectively. These results demonstrate that the system can be customized to fit the required operating conditions. Next, the specificity was investigated by exposing the signal-on system to non-target RNAs. Because we used miR-141 as the target RNA activator, various miRNAs were utilized as non-target RNAs. The results in Figure 3b show that the system is only responsive to the target activator (miR-141), generating a strong fluorescence signal without interference from other non-target RNAs.

### 3.4. Feasibility of the Signal-Off System

**Figure 4:**
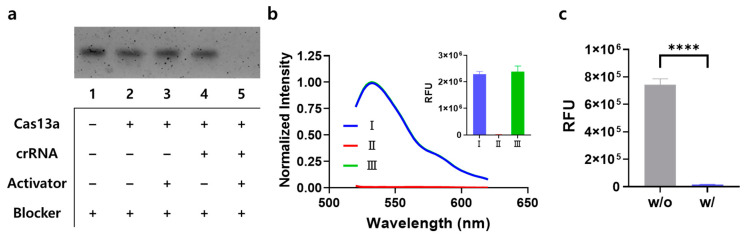
Feasibility of the signal-off system. (**a**) Agarose gel electrophoresis results confirming the Cas13a-catalyzed trans-cleavage reaction on blocker RNA. Lane 1: blocker RNA (300 nM), lane 2: lane 1 + Cas13a (5 nM), lane 3: lane 2 + RNA activator (miR-141; 1 nM), lane 4: lane 2 + crRNA (2.5 nM), and lane 5: lane 4 + RNA activator (1 nM). (**b**) Normalized fluorescence emission spectra and fluorescence intensity data proving that the blocker RNA mediated the activation of T7 RNAP suppressed by the T7 RNAP aptamer. The T7 RNAP-catalyzed transcription was performed as follows: (I) without the T7 RNAP aptamer, (Ⅱ) without the blocker RNA, and (Ⅲ) with all elements added. Fluorescence intensities were normalized against the maximum value. (**c**) Fluorescence intensities of the signal-off system based on the combination of Cas13a with T7 RNAP. “w/o” and “w/” indicate without and with target RNA activator, respectively. Unpaired *t*-test: **** *p* < 0.0001. All tests were performed with three technical replicates.

Similar to the signal-on system, a stepwise experiment was conducted to confirm the feasibility of the signal-off system. First, Cas13a-catalyzed trans-cleavage of the blocker RNA was demonstrated by gel electrophoresis analysis. Figure 4a shows that the gel band corresponding to the blocker RNA disappeared when the Cas13a-crRNA complex was present along with the RNA activator, verifying that the Cas13a-crRNA complex exerted effective trans-cleavage activity on the blocker RNA. Subsequently, the blocker RNA-mediated activation of T7 RNAP-catalyzed transcription suppressed by the T7 RNAP aptamer was investigated by measuring the fluorescence emission spectra of To1-biotin (Figure 4b). In accordance with the results of the signal-on system (Figure 2b), T7 RNAP-catalyzed transcription producing the Mango aptamer generated a high fluorescence signal because of the absence of the T7 RNAP aptamer (I), whereas the presence of the T7 RNAP aptamer and absence of the blocker RNA resulted in a negligible fluorescence signal (II). However, the addition of a blocker RNA that hybridizes to the T7 RNAP aptamer enables T7 RNAP to catalyze transcription without any problems, resulting in a high fluorescence signal (III) comparable to that in the absence of the T7 RNAP aptamer (I). Finally, we verified the feasibility of the signal-off system, which integrates two non-communicable reactions using Cas13a and T7 RNAP (Figure 4c). In the presence of the RNA activator, activated Cas13a catalyzed the trans-cleavage reaction on the blocker RNA, which allows the T7 RNAP aptamer to inhibit the transcription reaction, leading to a significantly lower fluorescence signal than that in the absence of the RNA activator. Furthermore, the blocker concentration was optimized to be 150 nM, which generated the highest signal-to-noise ratio in the presence of the RNA activator (Figure S2).

### 3.5. Performance of the Signal-Off System

**Figure 5:**
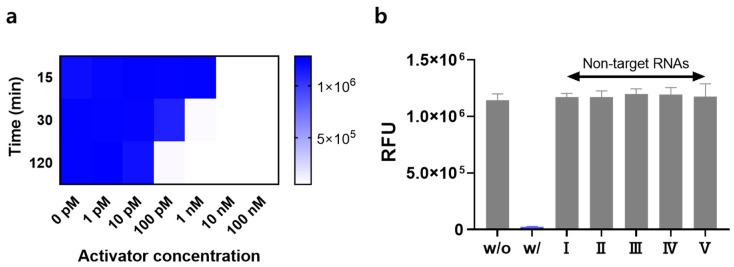
Performance of the signal-off system. (**a**) Fluorescence intensities measured at various activator concentrations (0–100 nM) and Cas13a reaction times (15–120 min). (**b**) Fluorescence intensities in the presence of target RNA activator and non-target RNAs (10 nM). All miRNA concentrations are 10 nM. “w/o” and “w/” indicate without and with target RNA activator, respectively. Non-target RNAs I–V are miR-21, miR-155, miR-182, miR-373, and miR-375, respectively. All tests were performed with three technical replicates.

Under optimal conditions, we conducted experiments to verify the detection performance of the signal-off system. Similar to the signal-on system, the Cas13a reaction time varied and the concentration of the RNA activator detectable by the system changed (Figure 5a). At a Cas13a reaction time of 30 min, the high fluorescence signal began to decrease at an RNA activator concentration of 1 nM. Increasing or decreasing the reaction time to 120 or 15 min changed the transition concentration; the high signal turned into a low signal of 100 pM or 10 nM, respectively. Notably, the detection range could be tuned by controlling the reaction time of Cas13a. Specificity experiments were also performed in the presence of non-target RNAs. Figure 5b shows that various non-target RNAs, such as miR-21, miR-155, miR-182, miR-373, and miR-375, generated a high fluorescence signal comparable to that in the absence of the target RNA activator, whereas the presence of the target RNA activator (miR-141) only led to a negligible fluorescence signal, confirming the high specificity of the proposed signal-off system.

### 3.6. Operation of the Aptamer-Based Switching System in Fetal Bovine Serum Samples

To confirm the practical applicability of our system to biological matrices, we attempted to operate the signal-on and -off systems in fetal bovine serum (FBS) [33,34]. Figure S3 shows that the presence of an RNA activator led to the high fluorescence signal in the signal-on system, whereas the presence of an RNA activator generated the low fluorescence signal in the signal-off system. Notably, the fluorescence signals of both signal-on and -off systems even in 10% FBS were comparable to those under pure conditions without 10% FBS, indicating that our aptamer-based switching system is compatible with biological matrices and can be utilized for more versatile applications.

## 4. Conclusions

In this study, we propose a switching system for the communication between non-interacting proteins using an aptamer as a network mediator. By employing a T7 RNAP aptamer that binds to T7 RNAP with high affinity and specificity, Cas13a and T7 RNAP with distinct catalytic activities were integrated to construct a signal-on system. Furthermore, a signal-off system was devised by rationally designing a blocker RNA that hybridizes to the T7 RNAP aptamer. To validate the proposed switching system, we carried out a systematic investigation which includes the confirmation of (i) the trans-cleavage reaction of Cas13a on both the T7 RNAP aptamer and the blocker RNA, (ii) the inhibitory ability of the T7 RNAP aptamer against T7 RNAP, and (iii) the operation of the integrated system. Importantly, the responsiveness of the system to the target RNA activator can be modulated by adjusting the Cas13a reaction time, and the system exhibits good specificity for the target RNA activator. The proposed system can be adapted for use with any RNA activators based on the design of an appropriate crRNA sequence. It is also expected to be used in combination with other signaling methods (e.g., field effect transistors) [35,36,37]. However, there is still room for improvement. For example, the construction of a reversibleswitching system, its application in the in vivo environment, and the toxicity evaluation in model organisms remain to be performed in future studies. Nonetheless, we believe that this study lays the foundation for the generation of an aptamer-based switching system that can contribute to the development of complex networks.

## Data Availability

The data presented in this study are available on request from the corresponding author.

## Supplementary Materials

The following supporting information can be downloaded at: https://www.mdpi.com/article/10.3390/bios14010047/s1, Table S1: Oligonucleotide sequences used in this study; Figure S1: Optimization of the signal-on system by measuring the fluorescence intensities at various concentrations of (**a**) Cas13a and (**b**) crRNA and (**c**) different transcription times; Figure S2: Optimization of the signal-off system by measuring the fluorescence intensities at various blocker RNA concentrations; Figure S3: Operation of the signal-on (**a**) and -off (**b**) systems in fetal bovine serum (10%). Reference [38] is cited in the Supplementary Materials.
