# Supplementary material for: Aptamer-Based Switching System for Communication of Non-Interacting Proteins
Source: Biosensors (Basel). 2024 Jan 16;14(1):47. doi: 10.3390/bios14010047 (PMC10812979; doi:10.3390/bios14010047)
Supplement: Supplementary file 1 [file biosensors-14-00047-s001.zip › biosensors-2805064-SI.pdf]

Supplementary Information

Aptamer-Based Switching System for Communication of Non-Interacting Proteins

Younghyeon Kim †, Daehan Nam †, Eun Sung Lee, Seokjoon Kim, Byung Seok Cha and Ki Soo Park *

Department of Biological Engineering, College of Engineering, Konkuk University, Seoul 05029, Republic of Korea

* Correspondence: E-mail: akdong486@konkuk.ac.kr or kskonkuk@gmail.com; Tel./Fax: +82-2-450-3742

† These authors contributed equally to this work.

Table S1. Oligonucleotide sequences used in this study.

Name	Oligonucleotide sequences (5′→ 3′)
T7 RNAP aptamer [1]	AGGCGAGCGUAAGUCAAUUCCACUAUCAUUGCUGCAAGC
Blocker RNA [1]	GAAGCAGCAAUGAUAGUGGAAUUGACUUACGC
crRNA template (+)	TAATACGACTCACTATAGGGGACCACCCCAAAAATGAAGGGGACT AAAACCCATCTTTACCAGACAGTGTTA
crRNA template (-)	TAACACTGTCTGGTAAAGATGGGTTTTAGTCCCCTTCATTTTTGGGG TGGTCCCCTATAGTGAGTCGTATTA
T7 promoter	TAATACGACTCACTATAGGG
Mango template	GTACGACAACTACCCCATACCAAACCTTCCTTCGTACCCCTATAGT GAGTCGTATTA
Target activator (miR-141)	UAACACUGUCUGGUAAAGAUGG
Non-target RNA I (miR-21)	UAGCUUAUCAGACUGAUGUUGA
Non-target RNA II (miR-155)	UUAAUGCUAAUCGUGAUAGGGGUU
Non-target RNA III (miR-182)	UUUGGCAAUGGUAGAACUCACACU
Non-target RNA IV (miR-373)	GAAGUGCUUCGAUUUUGGGGUGU
Non-target RNA V (miR-375)	UUUGUUCGUUCGGCUCGCGUGA

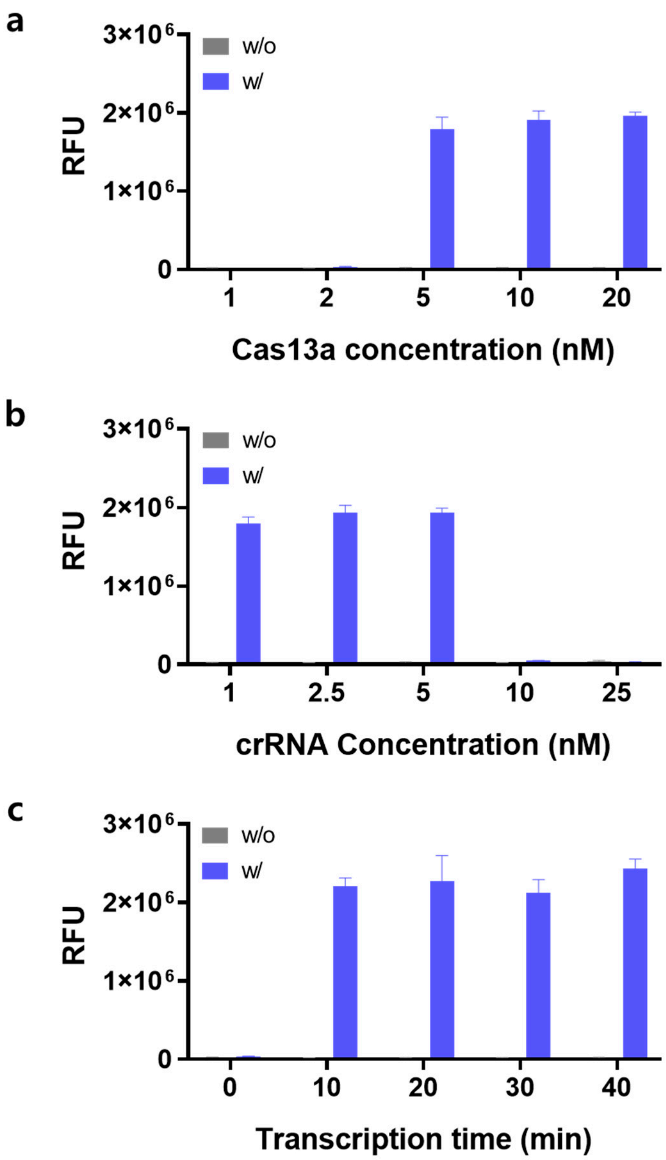

Figure S1. Optimization of the signal-on system by measuring the fluorescence intensities at various concentrations of (**a**) Cas13a and (**b**) crRNA and (**c**) different transcription times. "w/o" and "w/" indicate without and with target RNA activator (1 nM miR-141), respectively. All tests were performed with three technical replicates.

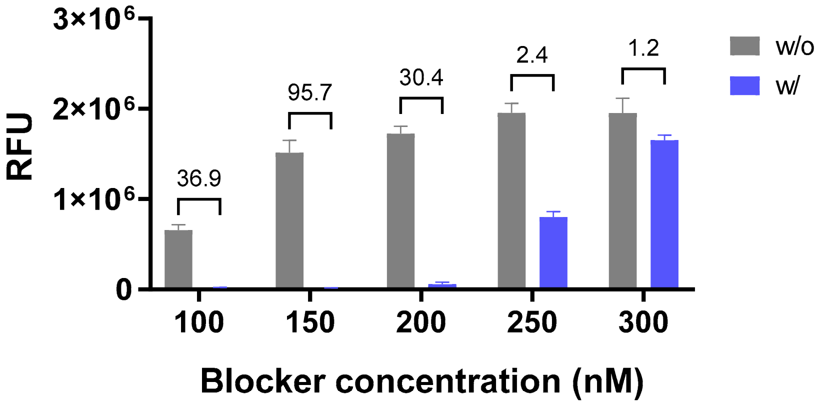

Figure S2. Optimization of the signal-off system by measuring the fluorescence intensities at various blocker concentrations. The fluorescence signal ratios (signal without target RNA activator divided by that with target RNA activator) are shown above each bar graph. "w/o" and "w/" indicate without and with target RNA activator (1 nM miR-141), respectively. All tests were performed with three technical replicates.

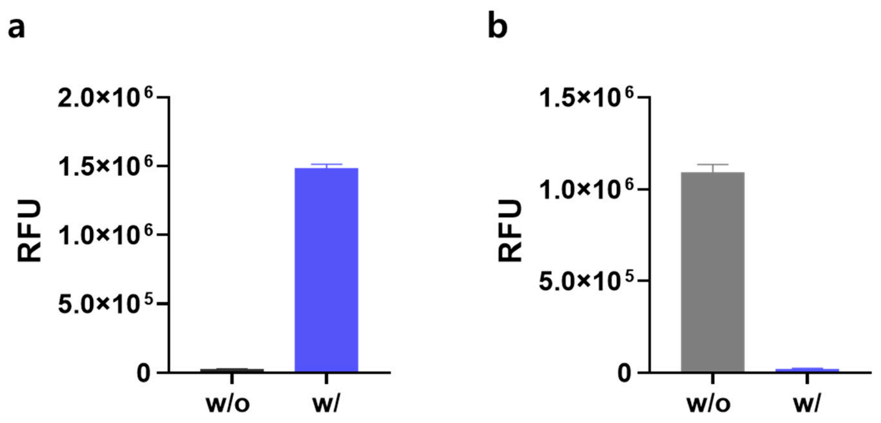

Figure S3. Operation of the signal-on (**a**) and -off (**b**) systems in fetal bovine serum (10%). "w/o" and "w/" indicate without and with target RNA activator (10nM miR-141), respectively. All tests were performed with three technical replicates.

Reference

1. Lloyd, J.; Tran, C.H.; Wadhwani, K.; Cuba Samaniego, C.; Subramanian, H.K.K.; Franco, E. Dynamic Control of Aptamer-Ligand Activity Using Strand Displacement Reactions. *ACS Synth. Biol.* **2018**, *7*, 30–37.
